# Supplementary material for: Learning curve and surgical outcome of robotic assisted colorectal surgery with ERAS program
Source: Sci Rep. 2022 Nov 29;12:20566. doi: 10.1038/s41598-022-24665-w (PMC9709162; doi:10.1038/s41598-022-24665-w)
Supplement: Supplementary file 1 — Supplementary Information 1. [file 41598_2022_24665_MOESM1_ESM.zip › LSC Raw data-Ñ[▒K/062/P_ñw╝╨░O▒Kñσ.pdf]

[redacted]

Pathologic diagnosis:
1. Colon, terminal ileum, and appendix, LAPA right hemicolectomy --- Adenocarcinoma, moderately differentiated, of ascending colon. Tumor invaded to mesocolic soft tissue and metastasized to mesocolic lymph nodes (2/26). The appendix, ileocecal valve, bilateral cut ends and radial margin are free of tumor.
2. Soft tissue, middle colic root, dissection --- Unremarkable lymph node (0/1).

Ancillary study for diagnosis:
IHC stains for MLH1, MSH2, PMS2 and MSH6 are done on section G. All above markers are preserved.

Prognostic and predictive factor:
1. Tumor size: 4x3.5 cm.
2. Depth of invasion: Mosocolic soft tissue.
3. Histologic grade: G2: Moderately differentiated.
4. Angiolymphatic invasion: Not identified.
5. Perineural invasion: Not identified.
6. Tumor deposits (discontinuous extramural extension): Not identified.
7. Surgical margins: a) Proximal Margin: Uninvolved by invasive carcinoma, distance of tumor from margin: 16 cm. b) Distal Margin: Uninvolved by invasive carcinoma, distance of tumor from margin : 4 cm. c) Radial or Mesenteric Margin: Uninvolved by invasive carcinoma, distance of tumor from margin: 3 cm.
8. Lymph node status: Involved (2/27).
9. Extranodal involvement: Not identified.
10. Tumor regression grading S/P CCRT: No known presurgical therapy.
11. Type of polyp in which invasive carcinoma arose: Not identified.
12. Additional pathologic findings: Not identified.
13. Pathological TNM Stage: pT3N1b (According to the eighth edition, 2017, American Joint Committee on Cancer Staging Guidelines for Tumors / 2017 CAP guideline).
14. TNM descriptors: Not applicable.

Gross description:
The specimen consists of 1) a segment of colon, 18 cm in length, and 7 cm in proximal circumference, 7 cm in distal cut end circumference, and a segment of terminal ileum, 6 cm in length, and 3 cm in cut end circumference. An unremarkable appendix, 6 cm, is noted. A gray tan firm annular elevated and centrally ulcerative tumor, 4x3.5 cm, is noted, 4 cm to the distal cut end. On cross section, the tumor invaded to the mesocolon with 3 cm to the radial margin grossly. The appendix, ileocecal valve, and both cut ends are free of tumor. 2) "middle colic root", a piece of yellow soft tissue, 4x3x2 cm. Representative part for section: A) specimen 2 B) proximal cut end C) distal cut end D) ileocecal valve E) appendix F) radial margin G-K) tumor L-N) lymph nodes.

Note:
1. The interpretation of MMR panel and recommended studies are as below: (1) No loss of nuclear expression of MMR proteins: low probability of microsatellite instability-high (MSI-H) . (2) Please note: There are exceptions to the above IHC interpretations. These results should not be considered in isolation, and clinical correlation with genetic counseling is recommended to assess the need for germline testing.
2. This case has been peer reviewed by two doctors.
#T-59420_2 #M-81403_2 2 1105 S0000F
#T-C4520_2 #M-81406_2 9 1105 000000
#T-59200_2 #M-09183_2 0 1105 000000

已電子簽章

敬告：本單僅供醫師參考使用，不具任何申請效力！！
請病患妥為保管，若不慎遺失需自負個人資料外洩責任！
